# Understanding Morning Emotions by Analyzing Daily Wake-Up Alarm Usage: Longitudinal Observational Study

**DOI:** 10.2196/50835

**Published:** 2024-11-29

**Authors:** Kyue Taek Oh, Jisu Ko, Nayoung ­Jin, Sangbin Han, Chan Yul Yoon, Jaemyung Shin, Minsam Ko

**Affiliations:** 1 Department of Human-Computer Interaction University of Hanyang Ansan, Gyeonggi-do Republic of Korea; 2 Department of Applied Artificial Intelligence University of Hanyang Ansan Republic of Korea; 3 Seoul Asan Medical Center Seoul Republic of Korea; 4 Delightroom Seoul Republic of Korea

**Keywords:** morning emotion, wake-up alarm usage, morning context, emotion monitoring, longitudinal observational study

## Abstract

**Background:**

Morning emotions can significantly affect daily wellness. While many studies have analyzed daily survey responses to identify factors influencing morning emotions, these methods require additional time and effort from individuals for emotional monitoring.

**Objective:**

This study aims to identify daily alarm usage patterns related to morning emotions.

**Methods:**

We recruited 373 users of the Alarmy app (DelightRoom) in the United States and South Korea and surveyed their demographics and usual behaviors related to morning emotions. Participants described their morning emotions over a 2-week period, during which we collected daily alarm app logs. We used a generalized estimating equation (GEE) method to identify factors affecting morning emotions.

**Results:**

The findings indicate that varied alarm usage is related to morning emotions. Alarm set time was positively associated with feelings of peacefulness and refreshment in the morning, while task-based alarms were related to nervousness. The time taken to deactivate the alarm after it rang was negatively correlated with happiness. In addition, usual behaviors and demographic factors were found to be related to morning emotions, consistent with previous studies.

**Conclusions:**

The study reveals that daily alarm usage is related to morning emotions, suggesting that daily alarm logs can supplement survey methods to facilitate daily emotion monitoring.

## Introduction

### Background

The mental or emotional state of an individual in the morning significantly impacts daily wellness and is associated with 6 dimensions of holistic health: physical, mental, spiritual, emotional, social, and environmental health [1]. Rothbard and Wilk [2] identified a correlation between employees’ morning moods and their emotional states throughout the day. Employees who began their day with positive emotions tended to maintain those emotions, positively influencing their interactions with customers. Conversely, morning fatigue adversely affects daily life by reducing the quantity and intensity of physical activities [3]. Studies suggest that daily performance can be enhanced by activities that positively influence one’s morning emotional state, such as listening to music [4] or taking a shower [5].

Numerous studies have attempted to understand and modify morning emotions. For instance, Sonnentag et al [6] reported that evening relaxation could promote positive emotional states the following morning. Nandy et al [7] found that regular exercise can enhance positive morning emotions. Among the various factors influencing morning mood, sleep quality is paramount. High-quality sleep is significantly associated with the suppression of negative emotions and the maintenance of positive emotions into the next day [8,9]. Early bedtimes have been shown to improve social interactions and emotional experiences [10]. Furthermore, the mood experienced in dreams can affect the emotional state the next morning [11].

However, to our knowledge, previous studies have not sufficiently addressed the impact of morning context—specific circumstances and environments experienced during the morning hours—on morning emotions. The morning period possesses unique characteristics, such as sleep inertia and preparation for daily routines, distinguishing it from other times of the day. Therefore, understanding awakening contexts could provide deeper insights into morning emotions. Furthermore, contextual factors are generally recognized as important in understanding specific behaviors [12-14]. Similarly, Oh et al [15] highlighted the necessity of considering morning contexts to comprehend changes in morning behavior.

### Objectives

This study aimed to explore the contextual factors that affect morning emotions. To achieve this, we analyzed alarm usage logs to evaluate the contextual information surrounding the moment of waking up. An alarm usage log reflects various aspects of waking behavior, such as the time and regularity of waking. In addition, monitoring and analyzing alarm usage logs is relatively easy because no additional user action is required. Previous studies to understand morning emotions have mainly relied on survey data that requires responses from the user. This study investigates the feasibility of a new method for monitoring morning emotions based on alarm logs by analyzing the relationship between alarm usage log data and morning emotions. If a significant relationship is found, this method could be expanded to monitor morning emotions using alarm usage log data. This approach could complement traditional methods that rely heavily on surveys, potentially providing a more efficient and unobtrusive way to understand and track morning emotions.

## Methods

### Participants

We recruited participants for this study from users of the Alarmy app (DelightRoom), focusing on the United States and South Korea, where the app is highly popular. Recruitment posts detailing the experiment’s background, objectives, and procedures, along with a web URL for the application, were disseminated through the app’s notice board and push notifications. In total, 373 subjects were recruited: 232 from the United States and 141 from South Korea.

The decision to use Alarmy as the focal application for this study was based on several compelling reasons. First, Alarmy is one of the most downloaded alarm apps globally, making it a widely used tool across diverse demographics and cultural contexts. Second, its unique features, such as mission-based alarms and loud alarm options, allow for a comprehensive analysis of wake-up behaviors and morning routines, providing insights that extend beyond basic alarm functions.

A key aspect of this study was to ensure that the findings were representative of the broader population of alarm users. To address potential concerns about generalizability, we compared the demographic characteristics of our sample to the larger Alarmy user population. The comparison revealed no significant differences in major usage statistics between our dataset and those used in previous studies analyzing large-scale Alarmy usage logs [16,17]. Furthermore, the age and gender distribution of our sample closely mirrored that of the overall Alarmy user base, suggesting that our findings are robust and applicable to a wide range of users.

Furthermore, a collaborative research agreement with Alarmy facilitated access to detailed log data, enhancing the robustness of the study. This agreement allowed us to leverage a large-scale dataset, ensuring that our sample was representative of the entire user base. Consequently, while the study exclusively recruited users of the Alarmy app, the demographic similarities between our sample and the broader population of Alarmy users support the generalizability of our conclusions.

All subjects participated voluntarily and received compensation of US $43 for their commitment. The compensation amount was carefully chosen to reflect the effort needed for daily participation over 2 weeks and the specific requirement to respond during morning hours. This ensured adequate participant motivation while minimizing any potential bias due to compensation. The study’s compensation strategy was designed to provide sufficient incentive to maintain participant engagement without compromising the integrity of the results. Evidence suggests that appropriately calibrated compensation is unlikely to bias experimental outcomes. According to a review by Singer and Couper [18], empirical studies typically find little evidence of payment influencing participants’ behavior in a way that significantly affects the results, provided that the payment is not coercively large. Our compensation of US $43 was benchmarked against typical study payments for similar durations and task demands, ensuring it was reasonable yet not overly influential.

### Experimental Procedure

We conducted a longitudinal observational study to explore the relationship between daily waking behaviors and morning emotions. Given that an individual’s demographics and characteristics could influence morning emotions, we categorized factors into three groups: (1) demographic, (2) usual, and (3) daily. Demographic factors included age and gender, while usual factors encompassed self-assessments of one’s typical morning state. Daily factors were derived from analyzing alarm app usage. As dependent variables, we specified 9 emotions commonly experienced in the morning—hopeful, happy, peaceful, refreshed, annoyed, tired, depressed, nervous, and neutral—along with a morning wellness score. These variables were measured through a presurvey, daily surveys, and alarm app usage logs.

The main experiment proceeded with participants first completing an 18-question presurvey covering demographics and their usual morning state. Over the course of 2 weeks, participants responded to daily surveys about their morning emotions and the time they went to sleep the previous night. These surveys were implemented and conducted using TypeForm, a web-based platform. To ensure accurate data collection on morning emotions, the initial survey link was sent 30 minutes after the participant’s alarm was set. If there was no response, a follow-up link was sent at 10 AM. This method aimed to capture participants’ emotions soon after waking while providing a second opportunity for those who missed the initial prompt. Alarm app usage data during the study period was shared for analysis.

After 2 weeks, the experimental procedure concluded, and participants were compensated for their involvement. This comprehensive approach, combining presurvey data, daily surveys, and usage logs, allowed for an in-depth analysis of the relationship between waking behaviors and morning emotions.

### Independent Variables

The independent variables in this study were classified into three categories: (1) demographic, (2) usual, and (3) daily. Table 1 provides detailed descriptions of each independent variable within these categories.

**Table 1:** Descriptions of independent variables.

Factors		Variable	Description
**Demographics**			
	Sex	demo_gender	Sex of the participant (male=0, female=1)
	Age	demo_age	Age of the participant
	Country	demo_country	Country of residence (United States=0, South Korea=1)
**Usual**			
	Morning state	usual_state_alert	Degree of alertness after waking up (Not at all alerts=1, Slightly alert=2, Fairly alert=3, Very alert=4)
	Morning state	usual_state_tired	Degree of tiredness after waking up (Very refreshed=1, Fairly refreshed=2, Slightly tired=3, Very tired=4)
	Morning state	usual_state_score	Usual morning condition, rated on a 1-7 scale, assessed at the start of the experiment.
	Habit	usual_habits_morningActivity	Habit of engaging in positive morning activities, such as exercise or meditation (No=0, Yes=1)
	Theory of Planned Behavior	usual_tpb_attitude	Attitude toward morning waking (Strongly disagree=1, Strongly agree=7)
	Theory of Planned Behavior	usual_tpb_ subjectiveNorm	Subjective norm toward morning waking (Strongly disagree=1, Strongly agree=7)
	Theory of Planned Behavior	usual_tpb_intention	Intention toward morning waking (Strongly disagree=1, Strongly agree=7)
	Theory of Planned Behavior	usual_tpb_control	Control toward morning waking (Strongly disagree=1, Strongly agree=7)
**Daily**			
	Alarm usage	daily_usage_dayOfWeek	Alarm usage on weekdays vs. weekends (weekday=0, weekends=1)
	Alarm usage	daily_usage_ringCount	Number of alarms used to wake up in the morning
	Alarm usage	daily_usage_ring2dismiss	Time elapsed from the alarm ringing to deactivation
	Alarm usage	daily_usage_continuity	Number of days the alarm was used in the past week
	Alarm usage	daily_first_ringTime	Time of the first alarm in the morning
	Alarm usage	daily_last_typeInBed	Percentage of wake-up tasks performed in bed after the last alarm
	Alarm usage	daily_last_typeOutOfBed	Percentage of wake-up tasks performed out of bed after the last alarm
	Alarm usage	daily_last_isLoud	Use of a loud sound for the last alarm
	Alarm usage	daily_last_isLabel	Use of a label for the last alarm
	Time to sleep	daily_sleep_time	Bedtime the previous night

### Demographic Factors

A total of 3 demographic variables were used: age, gender, and country, denoted as “demo_age,” “demo_gender,” and “demo_country,” respectively. Gender, being a categorical variable, was represented using a single dummy variable (male=0, female=1). Similarly, the participant's country was coded as a binary variable (United States=0, South Korea=1). All demographic variables were measured in a preliminary survey.

### Usual Factors

We examined variables related to the usual morning state through a presurvey, assessing three types of individual characteristics: (1) usual morning state, (2) habits, and (3) planned behavior.

Participants’ self-assessments of their usual morning state included aspects such as alertness and tiredness. These were measured using 4-point Likert scale questions adapted from the morning-evening wellness questionnaire [19]. Specifically, participants were asked, “How alert do you feel during the first half-hour after you wake up in the morning?” and “During the first half-hour after you wake up in the morning, how tired do you feel?” These responses were designated as “usual_state_alert” and “usual_state_tired,” respectively. In addition, participants rated their overall sense of well-being in the morning on a 7-point scale (“Rate your usual morning on a 7-point scale.”), which we denoted as “usual_state_score.”

We also considered the impact of participants’ morning habits on their emotions, as indicated by previous studies [7]. Participants reported their engagement in frequent dynamic activities (eg, exercise) or static activities (eg, meditation) in the morning. These activities were recorded as “usual_habits_morningActivity.”

Finally, we explored behavioral factors based on the theory of planned behavior (TPB) [20]. TPB variables are widely used to describe purposive human behaviors [21-23]. We developed a questionnaire with 4 questions on a 7-point Likert scale to evaluate participants’ usual target waking hours and morning behavioral patterns. The questions included: “Waking up on time in the morning is important to me,” “I intend to wake up on time in the morning,” “Other people think that I should wake up earlier in the morning,” and “I am confident that I am capable of waking up on time in the morning.” The responses to these questions were denoted as “usual_tpb_attitude,” “usual_tpb_intention,” “usual_tpb_subjectiveNorm,” and “usual_tpb_control,” respectively.

### Daily Factors

In this study, we analyzed the Alarmy app usage logs of participants during the experiment period to track their daily morning behavior. Given the reported differences in sleep patterns between weekdays and weekends [24], we denoted the days of the week with the variable “daily_usage_dayOfWeek,” coding weekdays as 0 and weekends as 1. Holidays were excluded from this classification. To examine the continuity of alarm use, the number of days the alarm was used in the previous week was recorded as “daily_usage_continuity.” In addition, we gathered information on how quickly users woke up in the morning. The number of alarms required to wake them up was denoted as “daily_usage_ringCount,” and the time from the ringing of the first alarm to its deactivation was denoted as “daily_usage_ring2dismiss.”

We further focused on the first and last alarms used in the morning. The time of the first alarm, indicating the targeted waking time, was marked as “daily_first_ringTime.” We also recorded the type of tasks required to dismiss the alarm. Alarms that could be deactivated without moving out of bed were indicated as “daily_last_typeInBed,” while those requiring movement out of bed were indicated as “daily_last_typeOutOfBed.” The use of a loud alarm sound for the last alarm was denoted as “daily_last_isLoud,” and the labeling of the last alarm was indicated by “daily_last_isLabel,” as the alarm label is the first text the user notices upon waking.

Finally, we included the time at which participants went to bed the night before. The ordinal variable “daily_sleep_time” was divided into 30-minute increments from 9 PM to 3 AM, with a total of 14 values numbered from 0 to 13.

### Dependent Variables: Morning Emotions

We collected overall condition scores on a 7-point scale each morning using the question, “Rate your morning on a 7-point scale,” and used these responses as the variable “daily_overall_state.” In addition, we considered 9 specific categories for morning emotions. Previous studies have defined emotions in various ways. For instance, Ekman [25] identified 6 basic emotions, while Plutchik [26] presented a detailed diagram of 8 core emotions. Russell [27] developed a circular emotion model using arousal and valence as the two axes. To specify the emotions people feel upon waking, we conducted a pilot survey with 29 participants before the main experiments. Respondents were asked to describe their feelings in the morning hours, and their responses were classified into 9 representative emotions: 4 positive emotions (hopeful, happy, peaceful, and refreshed) and 4 negative emotions (annoyed, tired, depressed, and nervous). The ninth category was “no emotion” (none).

### Analysis Methods

This study used generalized estimating equations (GEE) analysis [28] to effectively account for temporal changes in morning emotions. The GEE method is a widely used statistical technique for analyzing variables that are repeatedly measured over time [29,30]. While repeated-measures ANOVA is a common approach for analyzing repeated measurement data, the GEE method was deemed more appropriate for this study due to the presence of more than 2 independent variables.

### Ethical Considerations

To ensure participant confidentiality, all study data were deidentified. In addition, the experiment received an exemption for informed consent from the institutional review board of Hanyang University (HYUIRB-202205-011).

## Results

### User Statistics

Table 2 presents the descriptive statistics of the independent variables. Initially, we collected demographic information from participants based on 3 factors. The gender ratio was 49.6% (185/373 male participants). The average age of the participants was 23.94 (SD 9.65) years. Of the participants, 62.2% (232/373) resided in the United States, while the remainder lived in South Korea.

**Table 2:** Statistics of independent variables.

Factor		Variable	Mean (base)	SD
**Demographic**				
	Sex	demo_gender	0.49 (male)	0.50
	Age	demo_age	23.94	9.65
	Country	demo_country	0.62 (US)	0.48
**Usual**				
	Morning state	usual_state_alert	2.27	0.86
	Morning state	usual_state_tired	3.30	0.68
	Morning state	usual_state_score	4.29	1.39
	Habit	usual_habit_morningActivity	0.60	0.48
	Theory of planned behavior	usual_tpb_attitude	6.36	1.07
	Theory of planned behavior	usual_tpb_subjectiveNorm	5.72	1.62
	Theory of planned behavior	usual_tpb_intention	6.45	1.03
	Theory of planned behavior	usual_tpb_control	4.43	1.86
**Daily**				
	Alarm usage	daily_usage_dayOfWeek	0.79 (Weekday)	0.24
	Alarm usage	daily_usgae_ringCount	1.72	1.46
	Alarm usage	daily_usage_ring2dismiss	721.10	1496.27
	Alarm usage	daily_usage_continuity	3.78	1.67
	Alarm usage	daily_first_ringTime	25900.05	4570.43
	Alarm usage	daily_last_typeInBed	0.51	0.46
	Alarm usage	daily_last_typeOutOfBed	0.16	0.35
	Alarm usage	daily_last_isLoud	0.05 (True)	0.18
	Alarm usage	daily_last_isLabel	0.29 (True)	0.43
	Time to sleep	daily_sleep_time	7.55	3.06

We then examined usual lifestyle factors related to the morning state using a 7-point Likert scale. In the self-assessment of their usual morning condition within 30 minutes of waking, the overall health condition averaged 4.29 out of 7. The scores for alertness and tiredness were 2.27 and 3.30 out of 4, respectively. In addition, 60.3% (225/373) of participants reported that they typically engage in active exercises in the morning. The average scores for the four variables in the TPB category were as follows: attitude, 6.36; subjective norm, 5.72; intention, 6.45; and control, 4.43.

We also collected data on daily user behavior based on alarm use. Overall, 79% of the total usage occurred on weekdays, with participants using alarms an average of 3.787 days per week. On days when alarms were used, an average of 1.7 alarms were set due to participants either snoozing or setting multiple alarms. Approximately 12 minutes passed before deactivating the alarm. The average ring time of the first alarm of the day was 7:11:40 AM (25,900 seconds). The most commonly used alarm mission was the touch-a-button mission, with participants using this feature an average of 32.4% of the time. On average, 51.2% of wake-up tasks were performed in bed (eg, typing, math, memory, and shaking), while 16.3% of tasks required getting out of bed (eg, walking, squats, photo, and barcode). In addition, on average, participants selected loud alarm sounds 5.8% of the time and used alarm labels 29.9% of the time. Regarding usual bedtime, most respondents reported going to bed between 12 AM and 12:30 AM.

### GEE Analysis Results

Our GEE analysis identified unique wake-up context factors for each emotion. The comprehensive results of the GEE analysis are presented in Multimedia Appendix 1. In this section, we describe the variables that showed statistically significant results, including the odds ratio (OR), 95% CI of the original GEE coefficient values, and *P* values.

### Daily Overall State

Our analysis identified several variables significantly associated with the overall morning condition (daily_overall_state). Fewer alarms used per day (daily_usage_ringCount: OR 0.901, 95% CI –0.170 to –0.039; *P*=.002) and a greater number of days with alarm usage (daily_usage_continuity: OR 1.063, 95% CI 0.005-0.119; *P*=.03) were linked to higher overall scores. This suggests that consistency in alarm usage and reduced dependence on multiple alarms can enhance morning well-being.

The time of the first alarm (daily_first_ringTime) showed a positive correlation with the overall state (OR 1.133, 95% CI 0.052-0.199; *P*=.001), indicating that waking up later in the morning can contribute to a better overall morning state. Conversely, bedtime (daily_sleep_time) exhibited a negative relationship with the overall score (OR 0.743, 95% CI –0.370 to –0.224; *P*<.001), suggesting that going to bed earlier significantly improves morning well-being.

Furthermore, individuals who perceived their usual morning state positively (usual_state_score) tended to have better daily morning states (daily_overall_state) (OR 1.437, 95% CI 0.208-0.519; *P*<.001), highlighting the importance of a consistently good morning routine. Among the TPB variables, only attitude (usual_tpb_attitude) showed a positive correlation with the overall morning state of the day (OR 1.202, 95% CI 0.050-0.319, *P*=.007), implying that a positive outlook toward waking up is beneficial for morning well-being.

### Positive Emotions

**Figure 1:**
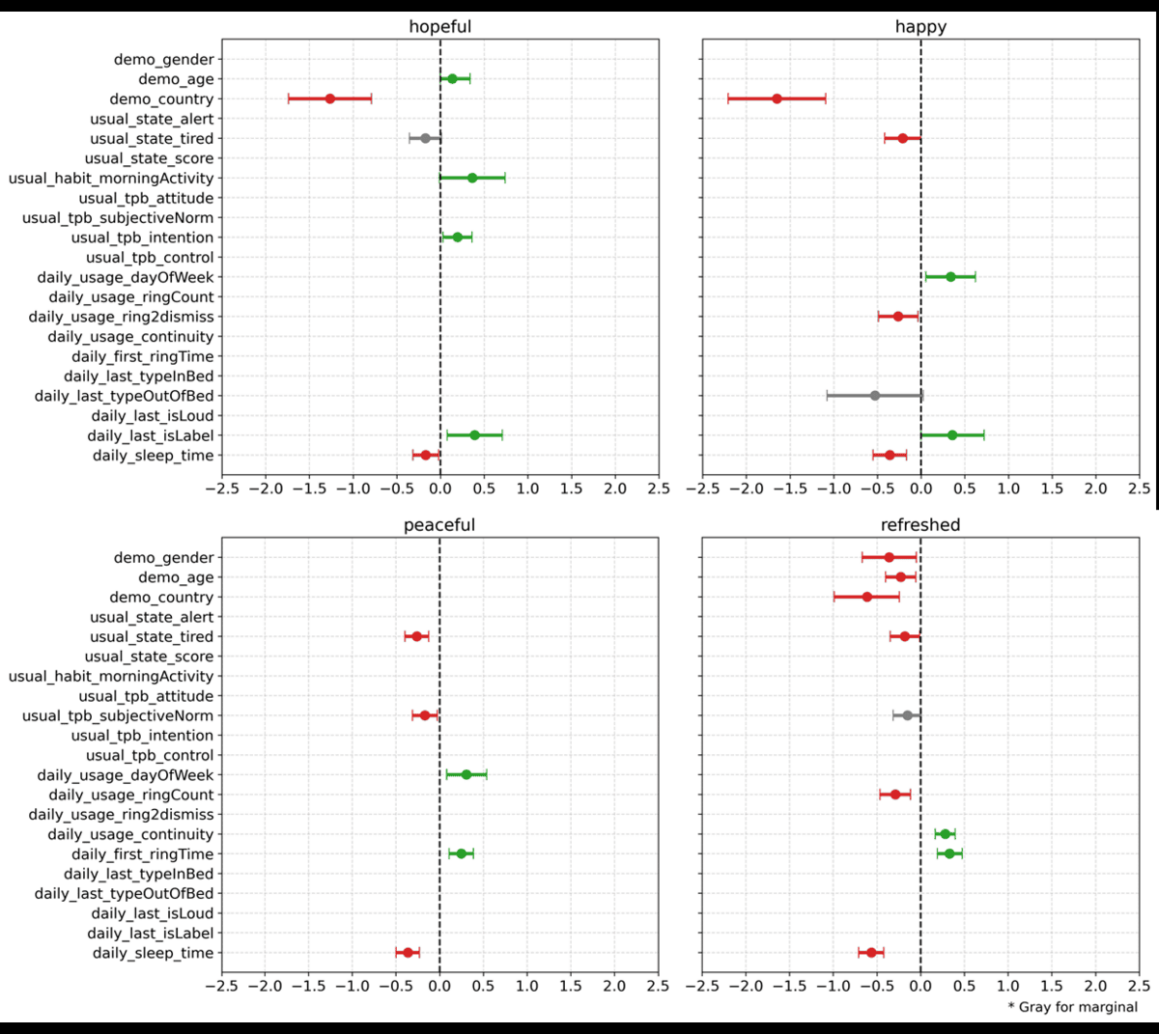
Results of generalized estimating equations analysis on positive emotions.

Figure 1 displays the GEE analysis results for positive emotions, including the β coefficient and CI for each significant independent variable.

#### Hopefulness

We identified several significant factors influencing the emotion of hopefulness in the morning. Using a label on the last alarm (daily_last_isLabel) increased the odds of feeling hopeful (OR 1.479, 95% CI 0.076-0.709; *P*=.01), suggesting that personalized alarm labels can positively impact morning emotions. Conversely, a late bedtime (daily_sleep_time) the previous day decreased the odds of feeling hopeful (OR 0.845, 95% CI –0.313 to –0.023; *P*=.02), indicating the importance of an early bedtime for a positive morning start. Age was positively correlated with hopefulness (demo_age: OR 1.181, 95% CI –0.004 to 0.339], *P*=.05), and Korean users had significantly lower odds of feeling hopeful compared with American users (demo_country: OR 0.282, 95% CI –1.738 to –0.789; *P*<.001). Feeling tired after waking (usual_state_tired) negatively influenced hopefulness (OR 0.842, 95% CI –0.353 to 0.011; *P*=.06), whereas engaging in physical activities in the morning (usual_habits_morningActivity) tended to increase hopefulness (OR 1.437, 95% CI –0.013-0.740; *P*=.05). Among the TPB factors, only intention (usual_tpb_intention) significantly increased the odds of feeling hopeful (OR 1.215, 95% CI 0.028-0.362; *P*=.02).

#### Happiness

The use of alarms significantly affected morning happiness. The elapsed time from the first alarm ring to its deactivation (daily_usage_ring2dismiss) was negatively associated with happiness (OR 0.768, 95% CI –0.489 to –0.036; *P*=.02), suggesting that quicker responses to alarms may improve morning mood. Alarm use on weekends (daily_usage_dayOfWeek) was 1.4 times more likely to increase happiness compared to weekdays (OR 1.402, 95% CI 0.054-0.623; *P*=.02). This may be because individuals who maintain a regular rhythm without distinguishing between weekdays and weekends tend to feel happier. Performing wake-up tasks out of bed after the last alarm (daily_last_typeOutOfBed) marginally decreased happiness (OR 0.590, 95% CI –1.077 to 0.023; *P*=.06), implying that remaining in bed for initial tasks might contribute to better mood. Using a label on the last alarm (daily_last_isLabel) was positively correlated with happiness (OR 1.430, 95% CI –0.002 to 0.719; *P*=.05), while a late bedtime (daily_sleep_time) significantly decreased happiness (OR 0.699, 95% CI –0.548 to –0.166; *P*<.001), highlighting the importance of an early bedtime for positive morning emotions. Morning tiredness (usual_state_tired) significantly reduced happiness (OR 0.809, 95% CI –0.418 to –0.005; *P*=.04), indicating the negative impact of fatigue on morning mood.

#### Peacefulness

Using alarms on weekends (daily_usage_dayOfWeek) was more likely to result in peaceful emotions in the morning compared to weekdays (OR 1.360, 95% CI 0.080-0.538; *P*=.008). Setting the alarm for a later time (daily_first_ringTime) was associated with increased feelings of peace (OR 1.281, 95% CI 0.109-0.388; *P*<.001). Like other positive morning emotions, a late bedtime (daily_sleep_time) was positively and significantly correlated with peacefulness (OR 0.696, 95% CI –0.497 to –0.229; *P*<.001). Those who usually felt tired upon waking (usual_state_tired) experienced less peace (OR 0.770, 95% CI –0.396 to –0.126; *P*<.001). In addition, participants who were more conscious of the people around them (usual_tpb_subjectiveNorm) tended not to feel peaceful (OR 0.845, 95% CI –0.310 to –0.026; *P*=.02), suggesting that social pressures may detract from morning peace.

#### Refreshed

The number of alarms used in a day (daily_usage_ringCount) negatively affected the likelihood of feeling refreshed (OR 0.748, 95% CI –0.464 to –0.117; *P*=.001). Regular alarm use (daily_usage_continuity) positively influenced feelings of refreshment (OR 1.324, 95% CI 0.167 to 0.395; *P*<.001). Early bedtimes (daily_sleep_time: OR 0.568, 95% CI –0.708 to –0.421; *P*<.001) and later wake-up times (daily_first_ringTime: OR 1.392, 95% CI 0.190-0.474; *P*<.001) were significantly associated with feeling refreshed. Younger users (demo_age) felt more refreshed (OR 0.796, 95% CI –0.399 to –0.055; *P*=.01), as did males (demo_gender: OR 0.698, 95% CI –0.669 to –0.050; *P*=.02) and American users (demo_country: OR 0.541, 95% CI –0.988 to –0.242; *P*=.001). Usual tiredness after waking (usual_state_tired) was negatively correlated with feeling refreshed (OR 0.835, 95% CI –0.349 to –0.011; *P*=.03). The subjective norm in TPB (usual_tpb_subjectiveNorm) marginally decreased the feeling of refreshment (OR 0.858, 95% CI –0.313 to 0.008; *P*=.06), indicating that awareness of others can negatively affect the feeling of refreshment.

### Negative Emotions

**Figure 2:**
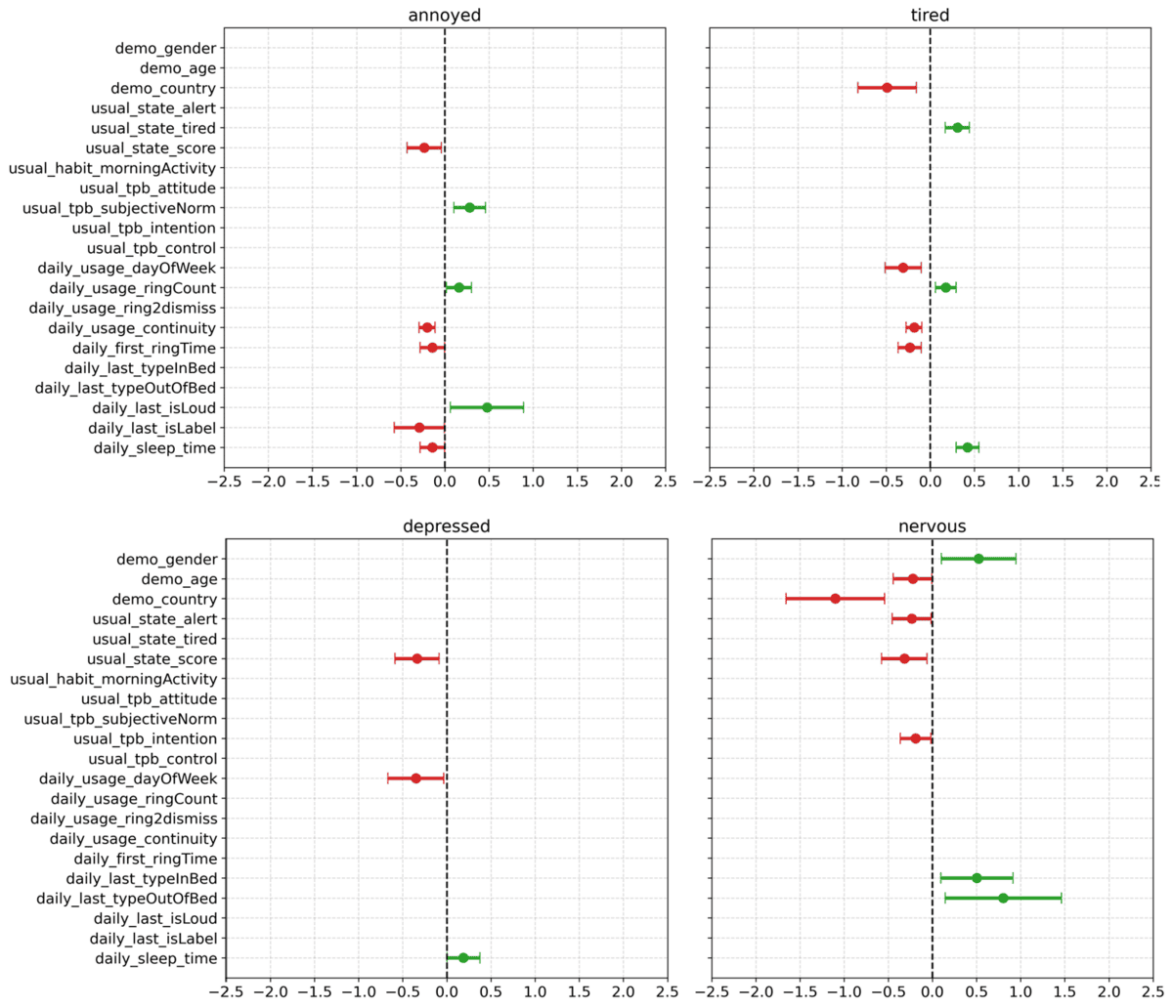
Results of generalized estimating equations analysis on negative emotions.

Our analysis identified several significant variables affecting negative morning emotions, as shown in Figure 2.

#### Annoyance

Participants who used alarms more frequently in a day (daily_usage_ringCount) were more likely to feel annoyed (OR 1.173, 95% CI 0.018-0.303; *P*=.02). Conversely, a higher number of consistent alarm use days (daily_usage_continuity) reduced the tendency for annoyance (OR 0.817, 95% CI –0.293 to –0.11; *P*<.001). Setting an alarm for a later time (daily_first_ringTime) was associated with decreased annoyance (OR 0.868, 95% CI –0.279 to –0.004; *P*=.04), suggesting that waking up later can improve mood. Loud alarm sounds (daily_last_isLoud) increased annoyance (OR 1.611, 95% CI 0.062-0.892; *P*=.02), whereas using alarms with label texts (daily_last_isLabel) was negatively related to annoyance (OR 0.748, 95% CI –0.573 to –0.007; *P*=.04). The need to wake up earlier (daily_first_ringTime) also increased annoyance (OR 1.221, 95% CI –0.279 to –0.004; *P*=.008). Participants’ usual morning state (usual_state_score) showed a negative correlation with morning annoyance (OR 0.791, 95% CI –0.427 to –0.041; *P*=.01). In addition, the subjective norm variable in TPB (usual_tpb_subjectiveNorm) was significantly and positively related to annoyance (OR 1.323, 95% CI 0.102-0.459; *P*=.002), suggesting that social pressures contribute to feelings of irritation.

#### Tiredness

Alarms used on weekends (daily_usage_dayOfWeek) resulted in significantly lower tiredness than alarms used on weekdays (OR 0.734, 95% CI –0.513 to –0.103; *P*=.003). More frequent alarm use in a day (daily_usage_ringCount) increased tiredness (OR 1.190, 95% CI 0.058-0.291; *P*=.003), whereas regular alarm use throughout the week (daily_usage_continuity) was associated with reduced tiredness (OR 0.831, 95% CI –0.274 to –0.096; *P*<.001). Later bedtimes (daily_sleep_time) increased the likelihood of feeling tired (OR 1.523, 95% CI 0.295-0.550; *P*<.001), as did earlier wake-up times (daily_first_ringTime: OR 0.792, 95% CI –0.365 to –0.101; *P*=.001). Participants in the United States felt significantly less tired in the morning compared with those in South Korea (demo_country: OR 0.612, 95% CI –0.822 to –0.158; *P*=.004). Those who reported usually feeling tired in the presurvey (usual_state_tired) were more likely to feel tired during the experiment (OR 1.357, 95% CI 0.168-0.444; *P*<.001), indicating a consistent pattern of fatigue.

#### Depression

Participants with lower scores on the usual morning state in the presurvey (usual_state_score) were more likely to feel depressed during the experiment (OR 0.713, 95% CI –0.588 to –0.088; *P*=.008). Using alarms on weekends (daily_usage_dayOfWeek) was negatively related to morning depression (OR 0.703, 95% CI –0.669 to –0.035; *P*=.02). Later bedtimes (daily_sleep_time) increased the likelihood of feeling depressed the following day (OR 1.205, 95% CI 0.002-0.373; *P*=.04).

#### Nervousness

Morning nervousness varied depending on the type of wake-up tasks performed. In-bed wake-up tasks (daily_last_typeInBed: OR 1.655, 95% CI 0.096-0.912; *P*=.01) and out-of-bed tasks (daily_last_typeOutOfBed: OR 2.229, 95% CI 0.146-1.460; *P*=.01) were both associated with increased nervousness, suggesting that the nature of the wake-up activity impacts stress levels. Usual morning state (usual_state_score: OR 0.727, 95% CI –0.575 to –0.061; *P*=.01) and usual waking level (usual_state_alert: OR 0.776, 95% CI –0.456 to –0.015; *P*=.03) were negatively related to nervousness. Participants in the United States were significantly more likely to feel nervous compared with those in South Korea (demo_country: OR 0.333, 95% CI –1.657 to –0.542; *P*<.001). Men were 1.68 times less likely to feel nervous than women (demo_gender: OR 1.687, 95% CI 0.101 to 0.946; *P*=.01), and younger users tended to feel more nervous (demo_age: OR 0.800, 95% CI –0.442 to –0.005; *P*=.04). Among the TPB factors, only intention (usual_tpb_intention) significantly decreased nervousness in the morning (OR 0.826, 95% CI –0.364 to –0.019; *P*=.02).

#### No Emotions

Our analysis identified a single variable significantly related to the absence of emotions in the morning. South Korean participants were significantly more likely to report no specific emotions upon waking compared to participants in the United States (demo_country: OR 4.083, 95% CI 0.859-1.956; *P*<.001). This finding may be influenced by cultural differences, as previous research has indicated that Western individuals tend to express their emotions more openly than their Eastern counterparts [31].

## Discussion

### Daily Alarm Usage Related to Morning Emotions

The findings of this study provide insightful correlations between mobile alarm usage and morning emotions. Several key factors influencing morning emotions were identified, and these results can be interpreted and applied to improve daily wake-up experiences.

First, the significant influence of alarm set time on morning emotions can be explained by the alignment with natural circadian rhythms. Waking up later allows individuals to complete their sleep cycles, leading to feelings of peacefulness and refreshment. Conversely, a late bedtime disrupts these cycles, suppressing positive emotions and augmenting negative ones. This underscores the importance of maintaining consistent sleep and wake times for emotional well-being. Similar results have been demonstrated in previous studies dealing with sleep duration and its effect on emotions. For example, Demasi et al [32] reported that sleep duration has a positive relationship with mental health. Insufficient sleep duration deteriorates emotional health conditions [33,34], such as depression [35]. Wrzus et al [36] identified the influence of the appropriate duration of sleep on mental health the following day by age. Also, a high quality of sleep fostered positive emotions on the morning of the next day [37].

Second, the method of alarm deactivation impacts morning emotions, likely due to the physical and cognitive demands placed on individuals immediately upon waking. Active wake-up tasks, such as taking pictures or performing squats, can induce stress and discomfort, resulting in nervousness and unhappiness. Previous study results show that those who do not wake up on time usually prefer hard alarms based on wake-up tasks [16]. These tasks are often preferred by individuals who struggle to wake up on time, further exacerbating their negative morning emotions.

Third, the detailed alarm settings, such as loudness and sound type, affect post-wake emotions due to their direct impact on sensory processing upon waking. Loud and jarring alarms can cause abrupt awakenings, leading to annoyance and a negative start to the day. Han et al [38] analyzed the effects of the types of alarm sounds on postwake emotions and noted that loud sounds could cause bad morning emotions. In contrast, using alarms with pleasant sounds and moderate volume can create a smoother transition from sleep to wakefulness. In addition, an alarm used on weekends had a significant effect on positive emotions, such as happiness and peace in the morning, while negative emotions, such as tiredness and depression, decreased.

Finally, the relationship between the wake-up process and morning emotions highlights the role of sleep inertia—a state characterized by reduced cognitive and sensory-motor performance after waking. A prolonged period between the alarm ringing and deactivation can prolong this inertia, decreasing morning happiness. Similarly, multiple alarms can increase annoyance and tiredness due to repeated disruptions in the waking process. This indicates that morning emotion can be related to sleep inertia [39], which refers to the transitional state experienced after waking and lowers cognitive and sensory-motor performance [40-42]. The findings of this study are consistent with previous study results that sleep inertia impairs positive emotions and encourages negative emotions [43,44]. In addition, using labeled alarms (eg, self-motivating to wake up on time or specifying the purpose) tended to induce feelings of hope and happiness and reduce annoyance in the morning.

Based on these interpretations, practical suggestions for optimizing alarm usage to improve morning emotions are as follows:

- Optimal alarm timing: Set alarms for later times in the morning, if possible, to align with natural circadian rhythms. Aim for consistent wake times that allow for the completion of sleep cycles, promoting peacefulness and refreshment.
- Consistent sleep schedule: Maintain a regular bedtime and wake time, even on weekends, to ensure sufficient sleep duration and prevent the suppression of positive emotions. A consistent sleep schedule supports overall emotional health and well-being.
- Gentle wake-up methods: Choose alarm deactivation methods that require minimal physical activity and are less intrusive. Avoid active tasks like taking pictures or performing exercises immediately upon waking, as these can increase stress and negative emotions.
- Moderate alarm volume and pleasant sounds: Use alarms with moderate volume and pleasant sounds to avoid abrupt awakenings and reduce annoyance. Select sounds that are calming and conducive to a gentle wake-up experience.
- Labeling alarms for motivation: Use labeled alarms with motivational messages or specified purposes to foster feelings of hope and happiness. This can make the wake-up process more intentional and positive.
- Minimize multiple alarms: Limit the number of alarms used in the morning to reduce repeated disruptions and minimize sleep inertia. Aim to wake up with a single alarm to enhance morning happiness and reduce tiredness.

In conclusion, while this study provides valuable insights into the relationship between alarm usage and morning emotions, future research with extended durations could offer a more comprehensive understanding. By adopting these practical suggestions, individuals can improve their morning emotional states and overall well-being.

### Nonalarm Factors Influencing Morning Emotions

Our study identified various nonalarm factors that influence morning emotions. First, demographic variables demonstrated significant differences in experiencing morning emotions. For example, male participants tended to be less nervous upon waking, consistent with previous studies indicating that females report more negative emotions than males [45,46]. In addition, significant correlations were found between age and feelings of nervousness and hope in the morning, aligning with previous findings [47]. Older participants reported lower levels of feeling refreshed upon waking, which may be related to decreased sleep quality with age [48]. Participants in the United States experienced a wider range of morning emotions compared to Korean participants, highlighting the need to consider cultural factors in studies of human emotion [47].

The usual morning states reported by participants also explained their morning emotions. For instance, alertness and tiredness within the first 30 minutes of waking were significantly related to negative emotions. Every negative emotion, except tiredness, tended to decrease when the usual morning score was higher. These findings align with previous studies [49,50], which show that individuals with low self-assessment scores tend to have lower quality of life in terms of physical and mental health, experiencing higher levels of pain, depression symptoms, and lower daily activity scores.

Previous research has emphasized the importance of behavioral patterns and lifestyles in understanding human emotions [51-53]. For example, Peluso and Guerra de Andrade [54] examined the relationship between emotion and physical activity in improving mental health, while Penedo and Dahn [55] explored the benefits of physical activity on emotional well-being. Studies have also used apps or web-based services to investigate the effects of physical activity on emotions [56,57]. Similarly, our results indicated that participants who engaged in physical activities after waking experienced more positive emotions, such as happiness.

Finally, we found that TPB variables related to waking on time significantly affect morning emotions, corroborating previous studies on morning behavior change [15]. For example, a positive intention to wake up on time was positively related to hopeful emotions and negatively related to nervousness. This suggests that a strong willingness to wake up on time fosters feelings of hope and reduces nervousness in the morning. The subjective norm variable was significantly positively correlated with annoyance and negatively correlated with feelings of peace and refreshment. This indicates that relying on external cues to wake up can hinder positive morning emotions.

### Unexplored Influences and Future Research

In our study, we ventured beyond traditional research paradigms by exploring how waking context, particularly alarm usage behaviors, influences morning emotions. This approach is significant as it sheds light on specific daily behaviors that can substantially impact emotional states at the start of the day. Notably, our research highlights the role of the Alarmy app’s various functions, such as mission-based alarms, in shaping individuals’ initial emotional responses. These findings underscore the importance of considering practical daily interactions with technology when studying emotional dynamics.

However, our investigation did not encompass all possible variables that could influence morning emotions. Factors such as family relationships, work-life balance, and health conditions can significantly influence individuals’ morning emotions [7,58]. Research indicates that behaviors like physical activity, social interactions, diet, and medication compliance play a crucial role in shaping morning emotional states, particularly in populations with mental health issues and histories of chronic homelessness [59]. In addition, loneliness has been associated with lower positive affect and higher distressed affect throughout the day, emphasizing the impact of social relationships on emotional well-being [60]. Furthermore, work-related stress and exhaustion can lead to physical and mental health problems, affecting overall living conditions and emotional states [61].

Cultural factors also play a significant role in influencing the positivity or negativity of morning emotions. For example, individuals from different countries exhibit distinct morningness patterns, highlighting strong national differences in morningness scores and factorial structures [47]. Cultural factors between the US and South Korea can significantly influence emotions, as evidenced in various studies. The emotional tendencies of Korean women in the US and Korea were influenced more by personal characteristics than cultural differences [62]. In addition, Korean American adolescents often face challenges due to the clash between traditional Korean culture and Americanization demands, impacting their emotional well-being and behavior in school settings [63]. Differences in aesthetic perceptions between US and South Korean participants highlight how cultural contexts shape emotional responses to website aesthetics [64]. Furthermore, US-aided construction projects in South Korea post-Korean War influenced the sociopolitical context and emotional landscape of the region by promoting democratic citizenship, private enterprise, and the American way of life [65].

The cultural dimensions proposed by Hofstede [66] further illuminate the distinctions between collectivistic and individualistic societies, which can deeply influence emotional responses. In collectivistic societies, individuals prioritize social harmony and often avoid behaviors that might discomfort others. This cultural trait could influence morning emotions, as people may wake feeling more harmonious or stressed based on their perception of social obligations or conflicts. Conversely, in individualistic cultures, where personal autonomy and self-expression are valued, transgressions of social norms can induce feelings of guilt, impacting morning emotional states.

For future research, it would be pertinent to incorporate these variables into the study design. A broader examination that includes diverse family dynamics, parenting styles, and a more extensive cultural representation would likely enhance the understanding of morning emotional states. In addition, longitudinal studies could provide deeper insights into how consistent interactions with alarm features, like those offered by Alarmy, influence long-term emotional and psychological well-being.

Such an integrated approach would not only validate the findings from this initial study but also expand our understanding of the intricate web of factors that influence morning emotions. Future research could also explore the impact of technology-based interventions tailored to different cultural contexts to improve morning emotional states. This could lead to more targeted interventions aimed at enhancing emotional well-being right from the start of the day, considering both cultural and familial contexts.

### Limitations

This study presents several limitations that should be considered when interpreting the overall findings within the specific context in which the research was conducted.

First, this research examined factors related to morning emotions by analyzing the usage logs of the Alarmy app from 373 users across two countries, emphasizing the significance of the wake-up context. Previous studies have seldom addressed app usage logs or waking conditions in relation to morning emotions, making the use of actual log data notably uncommon. However, other known factors, such as family relationships, work-life balance, and health conditions, which also influence morning emotions, were not included in this study. The practical constraints of conducting research in real-world settings, as opposed to a laboratory environment, limited the consideration and control of all variables. Future studies could benefit from incorporating these additional factors to offer a more comprehensive understanding of morning emotions.

Second, this study used actual alarm usage logs and daily survey data collected over a 2-week period. Consequently, it did not capture long-term usage patterns. Further research is necessary to observe long-term effects or pattern changes. Despite the short duration, the study gathered daily data from 373 real users, yielding a substantial number of sample instances that reflect real-life scenarios. The findings from this study will be valuable for designing future long-term studies.

Third, while this study analyzed the relationships between various variables and morning emotions, it primarily focused on correlations rather than causations. This means that although significant associations between variables and morning emotions were identified, direct causal relationships cannot be conclusively determined. However, understanding these correlations can still be valuable. Depending on the nature of the variables, these correlations can provide useful insights for predicting morning emotions and potentially guiding interventions.

Finally, this study concentrated on the Alarmy app among mobile alarm applications. Alarmy is widely used globally and provides a variety of wake-up alarm functions, and its log data was accessible for analysis. Therefore, the findings of this study should be interpreted within the context of the Alarmy app. Future research comparing different types of wake-up alarm apps or methods could offer insights into varying user experiences and usage patterns.

### Conclusions

This study aimed to comprehensively understand morning emotions by examining the context of morning waking through alarm usage. We categorized morning emotions into 9 distinct categories and identified factors affecting each. Specifically, we analyzed daily alarm usage logs, including ring times and deactivation types. The GEE analysis results revealed several factors related to each emotion. In addition to traditional factors such as demographics and usual state, daily alarm usage played a crucial role in understanding morning emotions.

This study demonstrates the feasibility of using waking alarms to monitor daily emotions. Existing methods for monitoring emotions primarily depend on explicit user data, such as survey responses [67,68]. While some studies have proposed monitoring methods based on implicit data from mobile sensors [69,70] or social activities from social media (eg, Twitter) [71-73], their practical application may be limited due to sensitive data collection and complex analyses. We believe that the findings of this study contribute to the improvement of future emotion monitoring by illustrating the relationship between waking alarm usage and specific emotions.

## Appendix

Multimedia Appendix 1 Generalized estimating equations analysis results.

## Abbreviations

GEE: generalized estimating equations
OR: odds ratio
TPB: theory of planned behavior
