# Supplementary material for: Understanding Morning Emotions by Analyzing Daily Wake-Up Alarm Usage: Longitudinal Observational Study
Source: JMIR Hum Factors. 2024 Nov 29;11:e50835. doi: 10.2196/50835 (PMC11645505; doi:10.2196/50835)
Supplement: Multimedia Appendix 1 [file humanfactors_v11i1e50835_app1.docx]

## Multimedia Appendix 1: GEE Analysis Results

### I. Daily Overall State

|  | **Coef** | **Std Err** | **z** | **P** | **CI [0.025, 0.975]** | |
| --- | --- | --- | --- | --- | --- | --- |
| Intercept | 4.7991 | 0.121 | 39.592 | < 0.001 | 4.562 | 5.037 |
| demo_gender [T.Female] | 0.0552 | 0.105 | 0.524 | 0.6 | -0.151 | 0.262 |
| demo_age | 0.0235 | 0.051 | 0.458 | 0.647 | -0.077 | 0.124 |
| demo_country [T.kor] | 0.1212 | 0.128 | 0.946 | 0.344 | -0.13 | 0.372 |
| usual_state_alert | 0.0343 | 0.061 | 0.559 | 0.576 | -0.086 | 0.154 |
| usual_state_tired | -0.0668 | 0.062 | -1.077 | 0.281 | -0.189 | 0.055 |
| usual_state_score | 0.3631 | 0.079 | 4.58 | < 0.001 | 0.208 | 0.519 |
| usual_habit_morningActivity | -0.076 | 0.116 | -0.655 | 0.513 | -0.303 | 0.151 |
| usual_tpb_attitude | 0.1847 | 0.069 | 2.694 | 0.007 | 0.05 | 0.319 |
| usual_tpb_subjectiveNorm | 0.0564 | 0.055 | 1.028 | 0.304 | -0.051 | 0.164 |
| usual_tpb_intention | 0.005 | 0.06 | 0.083 | 0.934 | -0.112 | 0.122 |
| usual_tpb_control | 0.0941 | 0.068 | 1.389 | 0.165 | -0.039 | 0.227 |
| daily_usage_dayOfWeek | 0.0855 | 0.067 | 1.286 | 0.199 | -0.045 | 0.216 |
| daily_usage_ringCount | -0.1045 | 0.034 | -3.108 | 0.002 | -0.17 | -0.039 |
| daily_usage_ring2dismiss | -0.0305 | 0.036 | -0.85 | 0.395 | -0.101 | 0.04 |
| daily_usage_continuity | 0.062 | 0.029 | 2.132 | 0.033 | 0.005 | 0.119 |
| daily_first_ringTime | 0.1254 | 0.038 | 3.335 | 0.001 | 0.052 | 0.199 |
| daily_last_typeInBed | -0.1622 | 0.095 | -1.71 | 0.087 | -0.348 | 0.024 |
| daily_last_typeOutOfBed | 0.0225 | 0.128 | 0.176 | 0.86 | -0.228 | 0.273 |
| daily_last_isLoud | -0.1251 | 0.123 | -1.018 | 0.309 | -0.366 | 0.116 |
| daily_last_isLabel | -0.015 | 0.093 | -0.161 | 0.872 | -0.198 | 0.168 |
| daily_sleep_time | -0.2968 | 0.037 | -7.948 | < 0.001 | -0.37 | -0.224 |

### II. Positive Emotions

#### Hopefulness

|  | **Coef** | **Std Err** | **z** | **P** | **CI [0.025, 0.975]** | |
| --- | --- | --- | --- | --- | --- | --- |
| Intercept | -1.5452 | 0.198 | -7.823 | < 0.001 | -1.932 | -1.158 |
| demo_gender [T.Female] | 0.0095 | 0.189 | 0.05 | 0.96 | -0.361 | 0.379 |
| demo_age | 0.1676 | 0.087 | 1.916 | 0.055 | -0.004 | 0.339 |
| demo_country [T.kor] | -1.2634 | 0.242 | -5.222 | < 0.001 | -1.738 | -0.789 |
| usual_state_alert | 0.1033 | 0.089 | 1.158 | 0.247 | -0.072 | 0.278 |
| usual_state_tired | -0.171 | 0.093 | -1.843 | 0.065 | -0.353 | 0.011 |
| usual_state_score | -0.0323 | 0.118 | -0.274 | 0.784 | -0.264 | 0.199 |
| usual_habit_morningActivity | 0.3634 | 0.192 | 1.892 | 0.058 | -0.013 | 0.74 |
| usual_tpb_attitude | -0.214 | 0.12 | -1.783 | 0.075 | -0.449 | 0.021 |
| usual_tpb_subjectiveNorm | 0.0075 | 0.089 | 0.084 | 0.933 | -0.167 | 0.182 |
| usual_tpb_intention | 0.195 | 0.085 | 2.284 | 0.022 | 0.028 | 0.362 |
| usual_tpb_control | -0.0866 | 0.115 | -0.752 | 0.452 | -0.312 | 0.139 |
| daily_usage_dayOfWeek | 0.1138 | 0.137 | 0.831 | 0.406 | -0.155 | 0.382 |
| daily_usage_ringCount | -0.1319 | 0.079 | -1.671 | 0.095 | -0.287 | 0.023 |
| daily_usage_ring2dismiss | -0.1251 | 0.091 | -1.371 | 0.17 | -0.304 | 0.054 |
| daily_usage_continuity | -0.0459 | 0.058 | -0.792 | 0.428 | -0.159 | 0.068 |
| daily_first_ringTime | -0.0925 | 0.072 | -1.278 | 0.201 | -0.234 | 0.049 |
| daily_last_typeInBed | -0.1985 | 0.164 | -1.21 | 0.226 | -0.52 | 0.123 |
| daily_last_typeOutOfBed | -0.195 | 0.216 | -0.904 | 0.366 | -0.618 | 0.228 |
| daily_last_isLoud | 0.3158 | 0.244 | 1.294 | 0.196 | -0.163 | 0.794 |
| daily_last_isLabel | 0.3925 | 0.162 | 2.429 | 0.015 | 0.076 | 0.709 |
| daily_sleep_time | -0.168 | 0.074 | -2.278 | 0.023 | -0.313 | -0.023 |

#### Happiness

|  | **Coef** | **Std Err** | **z** | **P** | **CI [0.025, 0.975]** | |
| --- | --- | --- | --- | --- | --- | --- |
| Intercept | -1.3949 | 0.217 | -6.425 | < 0.001 | -1.82 | -0.969 |
| demo_gender [T.Female] | -0.0959 | 0.203 | -0.473 | 0.636 | -0.493 | 0.301 |
| demo_age | -0.0549 | 0.103 | -0.53 | 0.596 | -0.258 | 0.148 |
| demo_country [T.kor] | -1.6497 | 0.285 | -5.793 | < 0.001 | -2.208 | -1.092 |
| usual_state_alert | 0.1059 | 0.108 | 0.981 | 0.326 | -0.106 | 0.317 |
| usual_state_tired | -0.2114 | 0.105 | -2.006 | 0.045 | -0.418 | -0.005 |
| usual_state_score | 0.0837 | 0.152 | 0.55 | 0.582 | -0.215 | 0.382 |
| usual_habit_morningActivity | 0.0562 | 0.199 | 0.283 | 0.778 | -0.334 | 0.446 |
| usual_tpb_attitude | -0.1455 | 0.133 | -1.091 | 0.275 | -0.407 | 0.116 |
| usual_tpb_subjectiveNorm | -0.049 | 0.097 | -0.504 | 0.614 | -0.24 | 0.141 |
| usual_tpb_intention | 0.1743 | 0.1 | 1.745 | 0.081 | -0.021 | 0.37 |
| usual_tpb_control | -0.0707 | 0.137 | -0.516 | 0.606 | -0.339 | 0.198 |
| daily_usage_dayOfWeek | 0.3388 | 0.145 | 2.335 | 0.02 | 0.054 | 0.623 |
| daily_usage_ringCount | -0.1025 | 0.118 | -0.87 | 0.385 | -0.333 | 0.128 |
| daily_usage_ring2dismiss | -0.2623 | 0.116 | -2.27 | 0.023 | -0.489 | -0.036 |
| daily_usage_continuity | -0.0514 | 0.058 | -0.883 | 0.377 | -0.165 | 0.063 |
| daily_first_ringTime | 0.0953 | 0.087 | 1.099 | 0.272 | -0.075 | 0.265 |
| daily_last_typeInBed | -0.2799 | 0.193 | -1.453 | 0.146 | -0.657 | 0.098 |
| daily_last_typeOutOfBed | -0.527 | 0.281 | -1.877 | 0.06 | -1.077 | 0.023 |
| daily_last_isLoud | -0.0826 | 0.242 | -0.342 | 0.732 | -0.556 | 0.391 |
| daily_last_isLabel | 0.3586 | 0.184 | 1.947 | 0.051 | -0.002 | 0.719 |
| daily_sleep_time | -0.3572 | 0.097 | -3.667 | < 0.001 | -0.548 | -0.166 |

#### Peacefulness

|  | **Coef** | **Std Err** | **z** | **P** | **CI [0.025, 0.975]** | |
| --- | --- | --- | --- | --- | --- | --- |
| Intercept | -1.5136 | 0.17 | -8.897 | < 0.001 | -1.847 | -1.18 |
| demo_gender [T.Female] | 0.035 | 0.15 | 0.233 | 0.816 | -0.259 | 0.329 |
| demo_age | -0.1186 | 0.086 | -1.387 | 0.166 | -0.286 | 0.049 |
| demo_country [T.kor] | -0.1784 | 0.182 | -0.982 | 0.326 | -0.535 | 0.178 |
| usual_state_alert | 0.0369 | 0.075 | 0.489 | 0.625 | -0.111 | 0.185 |
| usual_state_tired | -0.2611 | 0.069 | -3.796 | < 0.001 | -0.396 | -0.126 |
| usual_state_score | 0.1526 | 0.106 | 1.437 | 0.151 | -0.055 | 0.361 |
| usual_habit_morningActivity | 0.2413 | 0.16 | 1.512 | 0.131 | -0.071 | 0.554 |
| usual_tpb_attitude | -0.1072 | 0.083 | -1.284 | 0.199 | -0.271 | 0.056 |
| usual_tpb_subjectiveNorm | -0.168 | 0.072 | -2.325 | 0.02 | -0.31 | -0.026 |
| usual_tpb_intention | 0.0231 | 0.084 | 0.277 | 0.782 | -0.141 | 0.187 |
| usual_tpb_control | -0.1333 | 0.105 | -1.266 | 0.205 | -0.34 | 0.073 |
| daily_usage_dayOfWeek | 0.3089 | 0.117 | 2.646 | 0.008 | 0.08 | 0.538 |
| daily_usage_ringCount | -0.0877 | 0.093 | -0.94 | 0.347 | -0.271 | 0.095 |
| daily_usage_ring2dismiss | -0.0584 | 0.086 | -0.675 | 0.499 | -0.228 | 0.111 |
| daily_usage_continuity | 0.0644 | 0.06 | 1.082 | 0.279 | -0.052 | 0.181 |
| daily_first_ringTime | 0.2484 | 0.071 | 3.482 | < 0.001 | 0.109 | 0.388 |
| daily_last_typeInBed | 0.0154 | 0.144 | 0.107 | 0.915 | -0.267 | 0.298 |
| daily_last_typeOutOfBed | -0.1058 | 0.18 | -0.587 | 0.557 | -0.459 | 0.247 |
| daily_last_isLoud | -0.1175 | 0.254 | -0.462 | 0.644 | -0.616 | 0.381 |
| daily_last_isLabel | 0.1248 | 0.147 | 0.849 | 0.396 | -0.163 | 0.413 |
| daily_sleep_time | -0.3628 | 0.068 | -5.297 | < 0.001 | -0.497 | -0.229 |

#### Refreshed

|  | **Coef** | **Std Err** | **z** | **P** | **CI [0.025, 0.975]** | |
| --- | --- | --- | --- | --- | --- | --- |
| Intercept | -1.1284 | 0.181 | -6.221 | < 0.001 | -1.484 | -0.773 |
| demo_gender [T.Female] | -0.3597 | 0.158 | -2.279 | 0.023 | -0.669 | -0.05 |
| demo_age | -0.2273 | 0.088 | -2.592 | 0.01 | -0.399 | -0.055 |
| demo_country [T.kor] | -0.6148 | 0.19 | -3.23 | 0.001 | -0.988 | -0.242 |
| usual_state_alert | 0.0187 | 0.081 | 0.231 | 0.817 | -0.14 | 0.177 |
| usual_state_tired | -0.18 | 0.086 | -2.093 | 0.036 | -0.349 | -0.011 |
| usual_state_score | 0.0672 | 0.097 | 0.696 | 0.487 | -0.122 | 0.256 |
| usual_habit_morningActivity | -0.1137 | 0.163 | -0.696 | 0.486 | -0.434 | 0.206 |
| usual_tpb_attitude | 0.1379 | 0.131 | 1.057 | 0.291 | -0.118 | 0.394 |
| usual_tpb_subjectiveNorm | -0.1528 | 0.082 | -1.864 | 0.062 | -0.313 | 0.008 |
| usual_tpb_intention | 0.0547 | 0.084 | 0.651 | 0.515 | -0.11 | 0.22 |
| usual_tpb_control | -0.0867 | 0.088 | -0.987 | 0.324 | -0.259 | 0.085 |
| daily_usage_dayOfWeek | 0.2338 | 0.13 | 1.795 | 0.073 | -0.021 | 0.489 |
| daily_usage_ringCount | -0.2906 | 0.089 | -3.276 | 0.001 | -0.464 | -0.117 |
| daily_usage_ring2dismiss | 0.104 | 0.073 | 1.422 | 0.155 | -0.039 | 0.247 |
| daily_usage_continuity | 0.2811 | 0.058 | 4.816 | < 0.001 | 0.167 | 0.395 |
| daily_first_ringTime | 0.3318 | 0.072 | 4.581 | < 0.001 | 0.19 | 0.474 |
| daily_last_typeInBed | 0.0018 | 0.177 | 0.01 | 0.992 | -0.344 | 0.348 |
| daily_last_typeOutOfBed | 0.256 | 0.238 | 1.075 | 0.282 | -0.211 | 0.723 |
| daily_last_isLoud | -0.4378 | 0.284 | -1.544 | 0.123 | -0.994 | 0.118 |
| daily_last_isLabel | 0.1172 | 0.179 | 0.656 | 0.512 | -0.233 | 0.467 |
| daily_sleep_time | -0.5644 | 0.073 | -7.721 | < 0.001 | -0.708 | -0.421 |

### III. Negative Emotions

#### A. Annoyance

|  | **Coef** | **Std Err** | **z** | **P** | **CI [0.025, 0.975]** | |
| --- | --- | --- | --- | --- | --- | --- |
| Intercept | -1.3218 | 0.189 | -6.996 | < 0.001 | -1.692 | -0.952 |
| demo_gender [T.Female] | -0.1804 | 0.163 | -1.105 | 0.269 | -0.501 | 0.14 |
| demo_age | -0.0375 | 0.089 | -0.424 | 0.672 | -0.211 | 0.136 |
| demo_country [T.kor] | -0.2186 | 0.208 | -1.051 | 0.293 | -0.626 | 0.189 |
| usual_state_alert | -0.1088 | 0.086 | -1.264 | 0.206 | -0.278 | 0.06 |
| usual_state_tired | 0.1548 | 0.107 | 1.452 | 0.147 | -0.054 | 0.364 |
| usual_state_score | -0.2345 | 0.098 | -2.381 | 0.017 | -0.427 | -0.041 |
| usual_habit_morningActivity | 0.2188 | 0.188 | 1.161 | 0.246 | -0.151 | 0.588 |
| usual_tpb_attitude | 0.008 | 0.095 | 0.084 | 0.933 | -0.178 | 0.194 |
| usual_tpb_subjectiveNorm | 0.2805 | 0.091 | 3.077 | 0.002 | 0.102 | 0.459 |
| usual_tpb_intention | 0.0569 | 0.1 | 0.568 | 0.57 | -0.14 | 0.253 |
| usual_tpb_control | 0.1151 | 0.104 | 1.106 | 0.269 | -0.089 | 0.319 |
| daily_usage_dayOfWeek | -0.171 | 0.128 | -1.332 | 0.183 | -0.423 | 0.081 |
| daily_usage_ringCount | 0.1605 | 0.073 | 2.201 | 0.028 | 0.018 | 0.303 |
| daily_usage_ring2dismiss | -0.0332 | 0.063 | -0.524 | 0.6 | -0.158 | 0.091 |
| daily_usage_continuity | -0.2016 | 0.047 | -4.308 | < 0.001 | -0.293 | -0.11 |
| daily_first_ringTime | -0.1412 | 0.07 | -2.012 | 0.044 | -0.279 | -0.004 |
| daily_last_typeInBed | -0.0039 | 0.157 | -0.025 | 0.98 | -0.312 | 0.304 |
| daily_last_typeOutOfBed | -0.0303 | 0.223 | -0.136 | 0.892 | -0.467 | 0.406 |
| daily_last_isLoud | 0.4773 | 0.212 | 2.255 | 0.024 | 0.062 | 0.892 |
| daily_last_isLabel | -0.2901 | 0.144 | -2.011 | 0.044 | -0.573 | -0.007 |
| daily_sleep_time | 0.2006 | 0.075 | 2.657 | 0.008 | 0.053 | 0.349 |

#### B. Tiredness

|  | **Coef** | **Std Err** | **z** | **P** | **CI [0.025, 0.975]** | |
| --- | --- | --- | --- | --- | --- | --- |
| Intercept | 0.4282 | 0.16 | 2.671 | 0.008 | 0.114 | 0.742 |
| demo_gender [T.Female] | 0.1671 | 0.135 | 1.236 | 0.216 | -0.098 | 0.432 |
| demo_age | 0.0286 | 0.075 | 0.379 | 0.704 | -0.119 | 0.176 |
| demo_country [T.kor] | -0.49 | 0.169 | -2.896 | 0.004 | -0.822 | -0.158 |
| usual_state_alert | -0.0448 | 0.073 | -0.615 | 0.539 | -0.187 | 0.098 |
| usual_state_tired | 0.3064 | 0.07 | 4.351 | < 0.001 | 0.168 | 0.444 |
| usual_state_score | -0.0699 | 0.086 | -0.813 | 0.416 | -0.238 | 0.099 |
| usual_habit_morningActivity | -0.0148 | 0.145 | -0.102 | 0.919 | -0.3 | 0.27 |
| usual_tpb_attitude | -0.032 | 0.066 | -0.485 | 0.627 | -0.161 | 0.097 |
| usual_tpb_subjectiveNorm | 0.0661 | 0.07 | 0.937 | 0.349 | -0.072 | 0.204 |
| usual_tpb_intention | -0.0262 | 0.077 | -0.343 | 0.732 | -0.176 | 0.124 |
| usual_tpb_control | 0.0277 | 0.084 | 0.329 | 0.742 | -0.137 | 0.193 |
| daily_usage_dayOfWeek | -0.308 | 0.105 | -2.94 | 0.003 | -0.513 | -0.103 |
| daily_usage_ringCount | 0.1744 | 0.06 | 2.926 | 0.003 | 0.058 | 0.291 |
| daily_usage_ring2dismiss | 0.0894 | 0.061 | 1.468 | 0.142 | -0.03 | 0.209 |
| daily_usage_continuity | -0.1848 | 0.045 | -4.07 | < 0.001 | -0.274 | -0.096 |
| daily_first_ringTime | -0.2327 | 0.067 | -3.459 | 0.001 | -0.365 | -0.101 |
| daily_last_typeInBed | 0.2332 | 0.144 | 1.62 | 0.105 | -0.049 | 0.516 |
| daily_last_typeOutOfBed | 0.101 | 0.179 | 0.564 | 0.573 | -0.25 | 0.452 |
| daily_last_isLoud | 0.0998 | 0.211 | 0.474 | 0.635 | -0.313 | 0.513 |
| daily_last_isLabel | 0.0866 | 0.152 | 0.569 | 0.57 | -0.212 | 0.385 |
| daily_sleep_time | 0.421 | 0.066 | 6.396 | < 0.001 | 0.292 | 0.55 |

#### C. Depression

|  | **Coef** | **Std Err** | **z** | **P** | **CI [0.025, 0.975]** | |
| --- | --- | --- | --- | --- | --- | --- |
| Intercept | -2.1543 | 0.269 | -8.005 | < 0.001 | -2.682 | -1.627 |
| demo_gender [T.Female] | 0.347 | 0.223 | 1.553 | 0.12 | -0.091 | 0.785 |
| demo_age | -0.0407 | 0.124 | -0.328 | 0.743 | -0.284 | 0.203 |
| demo_country [T.kor] | -0.3565 | 0.287 | -1.241 | 0.215 | -0.92 | 0.207 |
| usual_state_alert | -0.0913 | 0.109 | -0.841 | 0.4 | -0.304 | 0.122 |
| usual_state_tired | 0.2027 | 0.119 | 1.703 | 0.089 | -0.031 | 0.436 |
| usual_state_score | -0.338 | 0.128 | -2.647 | 0.008 | -0.588 | -0.088 |
| usual_habit_morningActivity | -0.3285 | 0.233 | -1.409 | 0.159 | -0.785 | 0.128 |
| usual_tpb_attitude | 0.1612 | 0.129 | 1.248 | 0.212 | -0.092 | 0.414 |
| usual_tpb_subjectiveNorm | 0.1137 | 0.138 | 0.827 | 0.408 | -0.156 | 0.383 |
| usual_tpb_intention | -0.1535 | 0.085 | -1.811 | 0.07 | -0.32 | 0.013 |
| usual_tpb_control | 0.0418 | 0.126 | 0.33 | 0.741 | -0.206 | 0.29 |
| daily_usage_dayOfWeek | -0.3519 | 0.162 | -2.177 | 0.029 | -0.669 | -0.035 |
| daily_usage_ringCount | 0.1336 | 0.084 | 1.585 | 0.113 | -0.032 | 0.299 |
| daily_usage_ring2dismiss | 0.0505 | 0.07 | 0.717 | 0.473 | -0.088 | 0.189 |
| daily_usage_continuity | -0.105 | 0.065 | -1.612 | 0.107 | -0.233 | 0.023 |
| daily_first_ringTime | -0.1005 | 0.084 | -1.199 | 0.23 | -0.265 | 0.064 |
| daily_last_typeInBed | 0.1688 | 0.205 | 0.825 | 0.41 | -0.232 | 0.57 |
| daily_last_typeOutOfBed | 0.1292 | 0.298 | 0.433 | 0.665 | -0.456 | 0.714 |
| daily_last_isLoud | -0.04 | 0.372 | -0.108 | 0.914 | -0.769 | 0.689 |
| daily_last_isLabel | -0.1658 | 0.208 | -0.799 | 0.425 | -0.573 | 0.241 |
| daily_sleep_time | 0.1876 | 0.095 | 1.984 | 0.047 | 0.002 | 0.373 |

#### D. Nervousness

|  | **Coef** | **Std Err** | **z** | **P** | **CI [0.025, 0.975]** | |
| --- | --- | --- | --- | --- | --- | --- |
| Intercept | -2.4099 | 0.273 | -8.832 | < 0.001 | -2.945 | -1.875 |
| demo_gender [T.Female] | 0.5234 | 0.215 | 2.429 | 0.015 | 0.101 | 0.946 |
| demo_age | -0.2238 | 0.111 | -2.009 | 0.045 | -0.442 | -0.005 |
| demo_country [T.kor] | -1.0995 | 0.285 | -3.864 | < 0.001 | -1.657 | -0.542 |
| usual_state_alert | -0.2356 | 0.113 | -2.093 | 0.036 | -0.456 | -0.015 |
| usual_state_tired | -0.0723 | 0.114 | -0.636 | 0.524 | -0.295 | 0.15 |
| usual_state_score | -0.3181 | 0.131 | -2.429 | 0.015 | -0.575 | -0.061 |
| usual_habit_morningActivity | 0.0288 | 0.224 | 0.129 | 0.898 | -0.41 | 0.468 |
| usual_tpb_attitude | 0.1861 | 0.129 | 1.445 | 0.148 | -0.066 | 0.438 |
| usual_tpb_subjectiveNorm | -0.0379 | 0.108 | -0.349 | 0.727 | -0.251 | 0.175 |
| usual_tpb_intention | -0.1915 | 0.088 | -2.177 | 0.029 | -0.364 | -0.019 |
| usual_tpb_control | 0.1852 | 0.141 | 1.312 | 0.19 | -0.092 | 0.462 |
| daily_usage_dayOfWeek | -0.1287 | 0.147 | -0.874 | 0.382 | -0.417 | 0.16 |
| daily_usage_ringCount | 0.0981 | 0.071 | 1.377 | 0.169 | -0.042 | 0.238 |
| daily_usage_ring2dismiss | 0.0047 | 0.08 | 0.059 | 0.953 | -0.152 | 0.161 |
| daily_usage_continuity | -0.1226 | 0.073 | -1.671 | 0.095 | -0.266 | 0.021 |
| daily_first_ringTime | -0.0862 | 0.103 | -0.834 | 0.404 | -0.289 | 0.116 |
| daily_last_typeInBed | 0.5042 | 0.208 | 2.421 | 0.015 | 0.096 | 0.912 |
| daily_last_typeOutOfBed | 0.8028 | 0.335 | 2.396 | 0.017 | 0.146 | 1.46 |
| daily_last_isLoud | 0.2953 | 0.313 | 0.943 | 0.346 | -0.318 | 0.909 |
| daily_last_isLabel | -0.384 | 0.228 | -1.683 | 0.092 | -0.831 | 0.063 |
| daily_sleep_time | 0.1537 | 0.099 | 1.55 | 0.121 | -0.041 | 0.348 |

### IV. No Emotion

|  | **Coef** | **Std Err** | **z** | **P** | **CI [0.025, 0.975]** | |
| --- | --- | --- | --- | --- | --- | --- |
| Intercept | -2.498 | 0.261 | -9.58 | < 0.001 | -3.009 | -1.987 |
| demo_gender [T.Female] | -0.0108 | 0.202 | -0.054 | 0.957 | -0.406 | 0.384 |
| demo_age | -0.0439 | 0.11 | -0.399 | 0.69 | -0.259 | 0.172 |
| demo_country [T.kor] | 1.4076 | 0.28 | 5.031 | < 0.001 | 0.859 | 1.956 |
| usual_state_alert | 0.0669 | 0.105 | 0.64 | 0.522 | -0.138 | 0.272 |
| usual_state_tired | 0.0871 | 0.123 | 0.707 | 0.48 | -0.154 | 0.329 |
| usual_state_score | 0.0667 | 0.099 | 0.674 | 0.5 | -0.127 | 0.261 |
| usual_habit_morningActivity | -0.223 | 0.234 | -0.953 | 0.34 | -0.681 | 0.235 |
| usual_tpb_attitude | 0.0853 | 0.112 | 0.759 | 0.448 | -0.135 | 0.305 |
| usual_tpb_subjectiveNorm | 0.0308 | 0.104 | 0.297 | 0.766 | -0.172 | 0.234 |
| usual_tpb_intention | 0.0085 | 0.114 | 0.074 | 0.941 | -0.214 | 0.231 |
| usual_tpb_control | 0.0071 | 0.13 | 0.055 | 0.956 | -0.248 | 0.263 |
| daily_usage_dayOfWeek | 0.0486 | 0.135 | 0.359 | 0.72 | -0.217 | 0.314 |
| daily_usage_ringCount | -0.1195 | 0.103 | -1.158 | 0.247 | -0.322 | 0.083 |
| daily_usage_ring2dismiss | -0.1394 | 0.09 | -1.556 | 0.12 | -0.315 | 0.036 |
| daily_usage_continuity | -0.0667 | 0.069 | -0.961 | 0.336 | -0.203 | 0.069 |
| daily_first_ringTime | -0.1186 | 0.1 | -1.191 | 0.234 | -0.314 | 0.077 |
| daily_last_typeInBed | -0.029 | 0.203 | -0.143 | 0.886 | -0.426 | 0.368 |
| daily_last_typeOutOfBed | 0.1275 | 0.24 | 0.531 | 0.596 | -0.344 | 0.599 |
| daily_last_isLoud | 0.1205 | 0.285 | 0.423 | 0.672 | -0.438 | 0.679 |
| daily_last_isLabel | -0.1637 | 0.178 | -0.919 | 0.358 | -0.513 | 0.185 |
| daily_sleep_time | 0.0148 | 0.093 | 0.158 | 0.874 | -0.168 | 0.198 |
